# 15q26 deletion in a patient with congenital heart defect, growth restriction and intellectual disability: case report and literature review

**DOI:** 10.1186/s13052-021-01121-5

**Published:** 2021-09-16

**Authors:** Yahya Benbouchta, Nicole De Leeuw, Saadia Amasdl, Aziza Sbiti, Dominique Smeets, Khalid Sadki, Abdelaziz Sefiani

**Affiliations:** 1 Department of Medical Genetics, National Institute of Health, Rabat, Morocco; 2 Laboratory of Human Pathology, Faculty of Sciences, Mohammed V University, Rabat, Morocco; 3 Department of Human Genetics, Radboud University Medical Center, Nijmegen, Netherlands; 4 Research Team in Genomics and Molecular Epidemiology of Genetic Diseases, Genomic Center of Human Pathologies, Medical School and Pharmacy, University Mohammed V, Rabat, Morocco

**Keywords:** 15q26 deletion, CHD, Array-CGH, Case report

## Abstract

**Background:**

15q26 deletion is a relatively rare chromosomal disorder, and it is described only in few cases. Patients with this aberration show many signs and symptoms, particularly pre- and postnatal growth restriction, developmental delay, microcephaly, intellectual disability and various congenital malformations.

**Case presentation:**

We report on a girl, 4 years old, of consanguineous parents, with a 15q26 deletion. Clinical manifestations included failure to thrive, developmental delay**,** microcephaly, dysmorphic facies with broad forehead, hypertelorism, narrowed eyelid slits and protruding columella. The patient also showed skeletal abnormalities, especially clinodactyly of the 5th finger, varus equine right foot and left club foot. Additionally, she had teething delay and divergent strabismus. Heart ultrasound displayed two atrial septal defects with left-to-right shunt, enlarging the right cavities. Routine cytogenetic analysis revealed a shortened 15q chromosome. Subsequent array analysis disclosed a terminal 9.15 Mb deletion at subband 15q26.1-q26.3. Four candidate genes associated with 15q26 deletion phenotype were within the deleted region, i.e. *IGF1R, NR2F2, CHD2* and *MEF2A.*

**Conclusion:**

We report on an additional case of 15q26 monosomy, characterized by array-CGH. Molecular cytogenetic analysis allowed us to identify the exact size of the deletion, and four candidate genes for genotype-phenotype correlation. 15q26 monosomy should be considered when growth retardation is associated with hearing anomalies and congenital heart defect, especially atrioventricular septal defects (AVSDs) and/or aortic arch anomaly (AAA).

## Introduction

15q26 monosomy can occur either as a de novo event leading to a pure deletion or as a consequence of ring chromosome 15 formation and unbalanced translocation. Up to now, 58 cases of pure deletion have been documented in the literature [1, 2]. The sub-bands involved in this rearrangement include many candidate genes responsible for common symptoms, especially pre and postnatal growth retardation [3], developmental delay, microcephaly. Other genes were involved in more particular features like congenital heart disease (CHD) [4], skeletal anomalies [5], diaphragmatic hernia [6], kidney anomalies [1] and seizures [7]. This variability could be assigned to the difference in breakpoint location and the size of the deleted fragment. Recently, several authors focused on some particular regions especially the report of Klasseens et al., which restricted the critical region for congenital diaphragmatic hernia (CDH) to 4 Mb at 15q26.1-q26.3 band. Thus, two annotated genes, namely *NR2F2* (MIM 107773) and *CDH2* (MIM 602119), were considered relevant for CDH [8]. Other genes have been reported as playing crucial role in pathogenesis of 15q26 deletions, particularly *IGF1R, CHD2, NR2F2*, involved respectively in growth restriction [3], neurodevelopmental disorders [9], and CHD [4].

Herein, we report a further patient with CHD, intellectual disability and failure to thrive. Array-CGH displayed a terminal 9.15 Mb deletion spanning 15q26.1-q26.3, four relevant disease genes, i.e. *IGF1R, NR2F2, MEF2A*, and *CHD2* were involved and are directly related to the clinical presentation of our case.

Until now, no patient had a deletion of this exact size, without similar works in the literature that already focused on the types of CHD assigned to the 15q26 deletion, or on other possible genotype-phenotype correlations.

## Case report

The proband, a four-year-old girl, came to our attention because of dysmorphic face and heart malformation. She was the only child of healthy, consanguineous parents. There were no health problems in the family or a history of miscarriages. She was born at term by cesarean section because of intrauterine growth restriction (IUGR) associated with oligoamnios. IUGR was noted since the fourth month of pregnancy without that maternal or placental causes have been identified. Her birth weight was 950 g (< 3rd centile). Clinical history was suggestive of and congenital hypotonia. Upon clinical examination, her weight was 8 kg (< 3rd centile), height 81 cm (< 3rd centile) and head circumference 43 cm (< 3rd centile). She had dysmorphic features including broad forehead, hypertelorism, narrowed eyelid slits, low set ears, protruding columella, and short neck. She also presented with skeletal abnormalities, especially clinodactyly of the 5th finger, right foot varus equine, left club foot, biphalangeal fifth finger, and widely-spaced toes. Additionally, she had teething delay and divergent strabismus. Chest X-ray showed dorsal scoliosis and enlarged cardiac silhouette with a cardiothoracic ratio of 70%. An echocardiogram displayed a left-to-right shunt with significant flow, presence of two atrial septal defects (ASD) of 10 mm and 7 mm in width, dilating the right heart cavities and the pulmonary artery trunk with normal right pressures no aortic arch anomalies were evidenced. Her bone age was 2 years at a chronological age of 4 years. Hematologic investigations at the age of 4 and half years old showed normal serum concentrations of calcium, T4, TSH, GH and IGF1. Ocular assessment and brainstem acoustic potential evaluation revealed divergent strabismus and sensorineural hearing loss, respectively. Further investigations including brain MRI, computed tomography of the brain and abdominal ultrasound were normal.

## Methods and results

### Cytogenetics

**Fig. 1:**
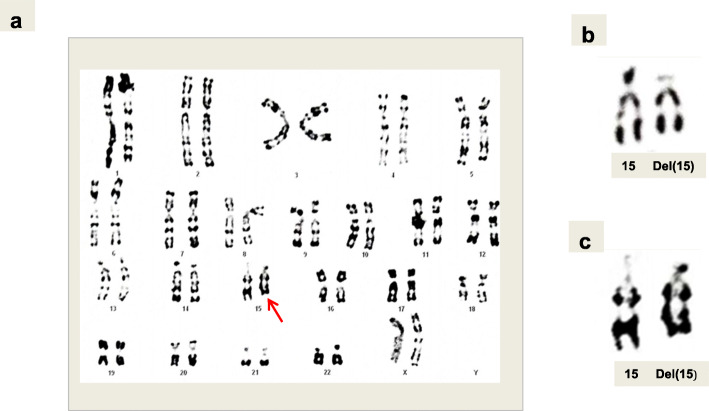
High-resolution R-banded karyogram (**a**,**c**), partial RHG (**b**) karyogram (**c**) showing deletion 15q chromosome with an abnormally short q-arm. (Red arrow)

Chromosome slides were prepared from cultured peripheral blood lymphocytes of the proband and her parents after obtaining informed consent. RHG-banding and high resolution R-banded chromosome analysis was performed on the three samples according to standard procedures. Both parents displayed normal karyotypes. Cytogenetic studies of the child showed an abnormal female karyotype with an apparently terminal deletion of the long arm of one chromosome 15 (Fig. 1). The patient’s karyotype was designated as 46,XX,?del (15q)dn.

### Array analysis

After disclosing the chromosomal aberration in the child, a genome wide array analysis was performed using the CytoScan HD SNP-based array platform (Affymetrix, Inc., Santa Clara, CA, USA) with an average resolution of approximately 20 kb following the manufacturer’s protocols. Inherent to the structure of the human genome, this resolution is not achieved for all regions such as the centromeric regions and heterochromatic parts.

**Fig. 2:**
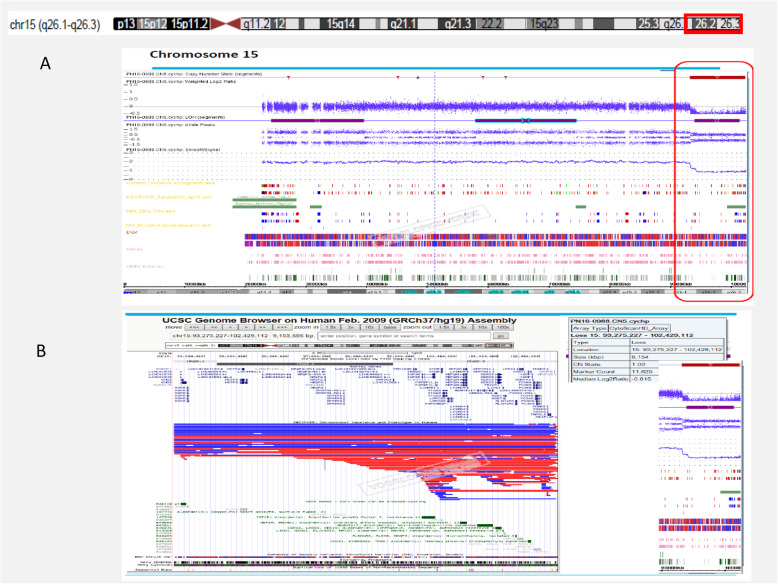
**A**. CGH-array data showing a terminal loss of 9.15 Mb in 15q26.1-q26.3 involving 93,275,228-102,429,113 breakpoints. **B**. UCSC genome browser assembly (GRCh37/hg19) highlighting genes involved in the loss segment

Genome wide array analysis confirmed the cytogenetic results and mapped the terminal deletion to a 9.15 Mb region encompassing 36 annotated genes with the proximal breakpoint at 93,275,228 Mb in band q26.1. (Fig. 2).

Based on these results, the final karyotype was designated as 46,XX,del [10](q26.1) dn.arr [GRCh37] 15q26.1q26.3(93,275,228-102,429,113) × 1.

In addition to this terminal loss of chromosome 15, several homozygous regions (ROH) were detected (164 Mb of the autosomal genome (~ 5.6%)), which is in agreement with the indicated consanguinity of the parents. The analysis of ROH using Genomic Oligoarray and SNP array evaluation tool (http://firefly.ccs.miami.edu/cgi-bin/ROH/ROH_analysis_tool_for_trial.cgi) did not reveal any candidate gene with recessive inheritance pattern.

## Discussion and conclusion

Here we report a further case of “**pure**” terminal deletion 15q26 associated with complex CHD. Fifteen other cases with such aberration and CHD were previously described. Table 1 summarizes clinical and cytogenetic data in these patients and ours.

**Table 1:** Clinical and cytogenetic data in patients with “pure” 15q26 deletion CHD

Clinical findings	Our case	Dateki 2011 [11]	Poot 2007 [10]	Tönnies 2001 [12]	Nakamua 2011 [4]	Slavotinek 2006 [6]		Hengstschlagr 2004 [13]	Bhakta 2005 [14]	Rump 2008 [15]	Choi 2011 [16]	Chui 2015 [17]	Biggio 2004 [18]	Okubo 2003 [19]	O’Riordan 2016 (38)	Iopez 2006 (39)
						Patient 1	Patient 2									
**Age**	4y	13y 9 m	8y 6 m	19 m	33 weeks	newoborn	newoborn	newborn	newborn	6 m	2 y	3 y	newborn	10 y	newborn	fetus/19 wg
**Gender**	F	F	F	F	F	F	F	F	F	M	M	F	F	F	M	F
**Position of 15q26 deletion**	15q26.1qter	15q26.2qter	15q26.2qter	15q26.1	15q26.2	15q26.2	15q26.2	15q26.1qter	15q26.1qter	15q26.2qter	15q26.2qter	15q26.2qter	15q26.1qter	15q26.1qter	15q26.2qter	15q26.1qter
**Deletion size**	9,15 Mb	5 Mb	6,87 Mb	NA	5,78 Mb	NA	NA	NA	NA	5.8 Mb	8.58 Mb	NA	NA	NA	6.554 Mb	NA
**Origin**	De novo	De novo	De novo	De novo	NA	De novo	NA	De novo	NA	De novo	NA	NA	NA	De novo	De novo	De novo
**IUGR**	+	–	+	+	+	NA	NA	+	+	–	+	NA	–	+	NA	+
**Microcephaly**	+	–	+	+	+	+	+	–	+	+	+	+	NA	+	+	NA
**Failure to thrive**	+	+	+	+	+	+	+	+	+	+	+	+	NA	+	+	NA
**Pschycomotor delay**	+	NA	+	+	+	NA	NA	NA	+	+	NA	+	NA	+	+	NA
**Intellectual disability**	+	+	–	NA	NA	NA	NA	NA	NA	+	NA	NA	NA	NA	NA	NA
**Facial dysmorphic features**	+	–	+	+	+	–	+	+	+	+	+	+	+	+	+	NA
**Broad nasal bridge**	+	–	–	+	NA	NA	+	NA	+	+	–	–	+	–	NA	NA
**Micrognathia**	+	–	+	+	+	NA	+	NA	NA	–	–	+	+	–	NA	NA
**Ear anomaly**	+	–	–	+	+	–	+	+	+	+	–	–	+	–	–	NA
**Eye anomaly**	+	–	+	–	NA	NA	NA	NA	NA	+	+	+	NA	+	NA	NA
**Cardiac defect**	+	+	+	+	+	+	+	+	+	+	+		+	+	+	+
**Hypoplastic heart**	–	–	–	–	–	+	–	–	–	–	–	+	–	–	–	–
**Enlarged heart**	+	–	–	–	–	–	–	–	–	–	–	–	–	–	–	–
**Cardiac shunt**	+	–	–	–	–	–	–	–	–	+	–	–	–	–	–	–
**Aortic arch anomaly**	–	–	–	+	–	+	+	+	+	–	+	–	+	–	+	–
**Ventricular septal defect**	–	+	–	+	+	+	+	+	+	–	–	–	–	+	–	–
**Patent ductus arteriosus**	–	–	–	+	–	–	+	–	+	–	–	–	–	–	–	–
**Atrial septal defect**	+	–	+	+	+	–	+	–	–	+	+	–	–	+	–	–
**Valvular defect**	–	–	+	–	–	+	+	–	+	–	–	–	+	–	–	–
**Lung hypoplasia**	–	–	–	–	–	+	+	–	+	–	–	–	+	–	–	–
**Diaphragmatic hernia**	–	–	–	–	–	+	+	+	–	–	–	–	+	–	–	+
**Kidney anomalies**	–	–	–	+	+	–	+	+	+	–	+	+	–	–	–	–
**Skeletal anomalies**	+	–	+	–	+	+	+	+	+	+	+	+	+	+	+	+
**Clinodactyly**	+	–	+	–	–	–	–	–	+	–	+	+	+	+	–	–
**Foot deformity**	+	–	–	–	–	+	–	+	+	+	–	–	–	+	–	–

Our proband shares many relevant signs and symptoms with other patients, especially pre- and postnatal growth retardation, developmental delay, skeletal anomalies, microcephaly, and hearing defects. In the other patients eye anomalies were observed less frequently. Less common features were found in some cases including kidney anomaly, CDH and lung hypoplasia; however, these are lacking in our patient. Through this table, we also note that the CHD was most often described as complex, with several concomitant abnormalities like in our patient. Among the major cardiac defect there are ASD/VSD and aortic arch anomaly. Valvulopathy, patent ductus arteriosus, cardiac shunt and hypoplastic heart were rarely described. Our patient shared some of these anomalies, namely ASD and cardiac shunt. However, she lacked VSD, AAA and valvular defect. Cardiomegaly was an unusual feature reported exclusively in our patient. Indeed, the atrial septal defect resulted in the formation of significant shunts, which led to volume overload of the right atrium and ventricle and consequently our patient developed cardiomegaly.

**Fig. 3:**
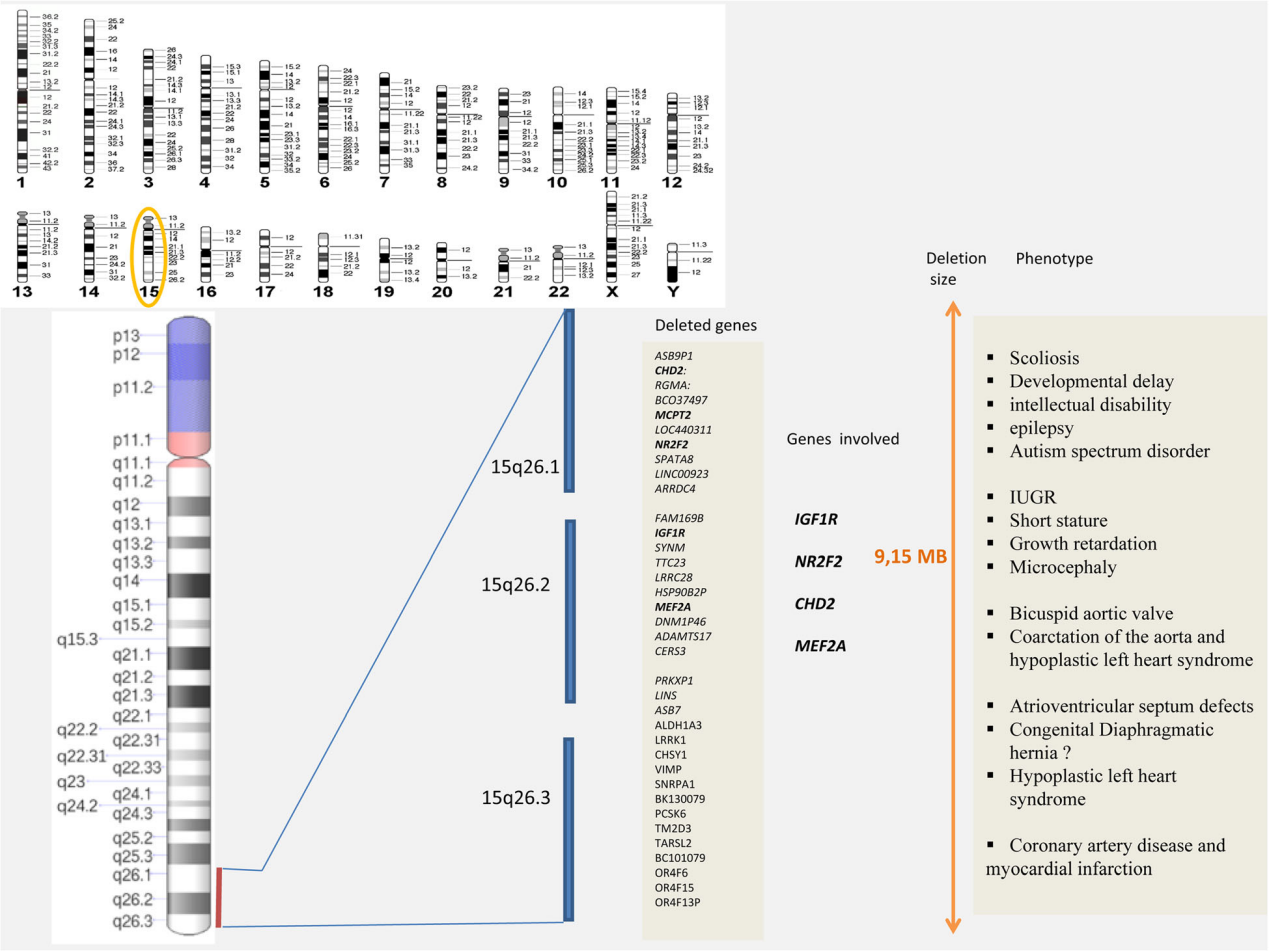
15q26.1-q26.3 deletions displaying the 36 missing genes together with the four genes involved in the Phenotype in our proband

Array analysis allowed us to characterize a 9.15 Mb deletion within the 15q26.1-q26.3 region. Comparable aberrations are often reported as de novo. Most often, terminal 15q deletions are found in combination with a terminal duplication of another chromosome due to an unbalanced translocation. To the best of our knowledge and according to the DECIPHER database, a deletion of this specific size has not been reported previously. Based on the Genome Data viewer (https://www.ncbi.nlm.nih.gov/genome/gdv/), the deleted segment encompasses 36 HGNC genes, 19 of them are referenced in the OMIM database, among which only *IGF1R, NR2F2, CHD2* and *MEF2A* are consistent with the phenotype described in our proband: (Fig. 3).

*IGF1R* (insulin like growth factor 1 receptor) (MIM 147370) lies on the 15q26.3 locus. It is bound to the growth factor ligands IGF1 and IGF2 to play a key role in pre- and post-natal development [20, 21]. The crucial impact of *IGF1R* on growth processes was underlined by the growth restriction found in individuals with pathogenic variants in the *IGF1R* gene [3, 22], in addition to patients with a 15q26 deletion leading to haploinsufficiency [23]. To the best of our knowledge, no heart anomalies have ever seen in patients carrying IGF1 or *IGF1R* mutations nor in knockout mice lacking these genes. Therefore, it is unlikely that the onset of CHD is only caused by haploinsufficiency of the *IGF1R* gene [4].

*NR2F2* (Nuclear *NR2F2* (Nuclear Receptor Subfamily 2, Group F, Member 2) (MIM 107773), located at 15q26.2 locus, is involved in angiogenesis and heart development [24], Indeed, *NR2F2* haploinsufficiency in patients with a 15q26 deletion appears to be associated with heart malformations [25]. In addition, variants within the *NR2F2* gene were found to cause non-syndromic atrioventricular septal defects (AVSDs) and other heart defects as well [26] . Moreover, this gene has been implicated to be involved in some patients with diaphragmatic hernia [6, 27], but this was not reported in others [28, 29] nor present in our patient.

*CHD2* (Chromodaine helicase DNA-binding protein) belongs to a family of ATP-dependant chromatin remodeling proteins involved in chromatin regulation [12]. Mutations in this gene were associated with severe non-syndromic intellectual disability [13], as well as epileptic encephalopathy [14]. Additionally, disruption of *CHD2* was associated with scoliosis in murine models [15]. Interestingly, this anomaly was observed in our patient as well as a few in the literature [9, 16, 28]. These findings together highlight the involvement of *CHD2* dysfunction in neurodevelopmental disorders and scoliosis. This gene has previously been proposed as a candidate gene for the CDH [8], but this was not evident in our case or elsewhere [7, 9]. *MEF2A* (Mads Box Transcription Enhancer Factor 2, Polypeptide A) (MIM 600660), mapped to the human chromosome 15q26.3 region, is member of the myocyte enhancer family of transcription factors (MEF2) [17]. The subunit *MEF2A* is expressed in endothelial and smooth muscle cells of coronary arteries. Subsequently, *MEF2A* mutations can disturb the growth or differentiation of these cells, increasing the risk of developing coronary artery disease (CAD)/ myocardial infarction (MI) [18, 19]. CAD/MI was not evident in patients with 15q26 deletion involving *MEF2A.* This could be explained by the relatively young age of these patients compared to others described by Wang and Bhagavatula whose age of diagnosis was between 36 and 80 years [18, 19]. Therefore, regular checking up would be useful from the third decade onwards in these patients.

To sum up, this work focused on the main genes whose haploinsufficiency could explain heart disease in patients with 15q26 monosomy, i.e. the *NR2F2* gene involved respectively in AVSDs and AAA/hypoplastic left heart. Scoliosis and psychomotor delay in our patient would be explained by the *CHD2* gene disruption. The phenotype in our patient could also be ascribed to the high rate of homozygous regions outlined by the CGH array, without excluding the possible contribution of epigenetic and environmental factors as well. 15q26 monosomy should be considered when growth retardation is associated with congenital heart defect (mainly AVSDs and/or AAA).

Patients with 15q26 deletion need a multidisciplinary management, which includes endocrinological assessment evaluating also possible GH therapy [30], as well as cardiologic, orthopedic and psychomotor follow-up. The genetic counseling in our family was delicate since the parents refused to undergo the array CGH analysis that is important for a complete familiar counseling.

## Data Availability

All data is contained in the manuscript.

## Abbreviations

CHD: Congenital Heart disease
CNV: Copy number variations
*NR2F2*: Nuclear Receptor Subfamily 2, Group F, Member 2
AVSDs: Atrioventricular septal defects
*MEF2A*: Mads Box Transcription Enhancer Factor 2, Polypeptide A
CAD/ MI: Coronary artery disease/ myocardial infarction
CoA: Coarctation of the aorta
*IGF1R*: Insulin like growth factor 1 receptor
ASD: Atrial Septal Defects
VSD: Atrioventricular Septal Defects
AAA: Aortic Arch Anomaly
IUGR: intrauterine growth restriction
CDH: congenital diaphragmatic hernia
